# Comparison the Effect of Metformin and Clomiphene Citrate on Sirtuin3 gene Expression in the Oocytes of Mice with Polycystic Ovary Syndrome

**DOI:** 10.22037/ijpr.2020.113321.14236

**Published:** 2020

**Authors:** Fatemeh Kamalipour, Hanieh Jalali, Mahnaz Azarnia

**Affiliations:** *Department of Animal Biology, Faculty of Biological Sciences, Kharazmi University, Tehran, Iran. *

**Keywords:** Oocyte, Fertilization, Sirtuin, Polycystic ovary syndrome, Metrormin, Clomiphene citrate

## Abstract

Oxidative stress (OS) is a common biological event in polycystic ovarian syndrome (PCOS), causing oocytes to undergo OS-induced changes. Sirtuin3 (Sirt3) has a critical role in oocyte maturation through the modulation of OS. In the current study, we compared the effects of metformin and clomiphene citrate on the expression of the Sirt3 gene in oocytes obtained from the mice, induced by PCOS. The induction of PCOS was performed by the single injection of estradiol valerate. The animals were divided into control, PCOS, metformin (500 mg/Kg), and clomiphene (18 mg/kg) groups. At the end of the experiment, the levels of LH and FSH were determined using the ELISA method. The ovarian tissues were evaluated histologically, and the expression of the Sirt3 gene was analyzed by the Real-time PCR. The induction of PCOS led to an increase in the ratio of LH/FSH elevation, the number of follicle atresia, as well as the presence of hydrated cysts. The results showed that both treatment regimens returned the altered parameters to the baseline values. The gene of Sirt3 was significantly (*P *< 0.001) reduced in the PCOS group compared to the control. Also, no significant difference was found in the expression of Sirt3 between clomiphene and PCOS group, whereas, in the metformin group, Sirt3 expression had the higher rate of expression in comparison with the PCOS group (*P *< 0.05). The administration of metformin and clomiphene showed that metformin is capable of preventing the downregulation of the Sirt3 gene in oocytes, collected from PCOS mice.

## Introduction

The Sirtuin family is a group of histone deacetylase proteins that contribute to the removal of the acetyl group from various proteins and post-translationally modify them (1). Since the NAD^+^/NADH ratio is an important factor in controlling the activity of the Sirtuin family, it seems that these proteins play an essential role in regulating cellular metabolism (2). Seven members of the Sirtuin family have been so far identified in mammals, each of which has a particular position and role within the cell (3). Sirtuin3 (Sirt3) is localized in mitochondria and has a critical role in the regulation of the acetylation process in most of the mitochondrial proteins (4). Sirt3 acts as a sensor for reactive oxygen species (ROS) and protects the cells against oxidative stress through activating antioxidant enzymes and also deacetylating the isocitrate dehydrogenase-2 enzyme (IDH2) (5, 6). Sirtuin3 is also involved in oocyte maturation, through the modulation of ROS (7). Studies have indicated that the inhibition of sirtuin3 via RNA interference can reduce the number of murine and porcine blastocysts, predominantly through the formation of ROS. In fact, the maternal heritage of the sirtuin3 gene is a critical factor in protecting the early embryo against oxidative stress (8, 9). 

Polycystic ovary syndrome (PCOS) is a common disorder affecting 6-20% of women in their reproductive age (10). Hirsutism, acne, obesity, irregular menstruation, and chronic anovulation are the most common complications which are manifested in patients (11). Metformin, clomiphene citrate (CC), flutamide, letrozole, spironolactone, and other medications are regularly prescribed to patients to manage the symptoms, early mentioned (12). Metformin and CC are considered first-line therapy to induce ovulation in infertile women (13). Metformin primarily induces ovulation by lowering the insulin level, thus inhibiting its negative effect on ovarian tissues. Clomiphene, as an estrogen receptor antagonist, increases the concentration of FSH, thereby stimulating the follicular growth phase and, ultimately, ovulation induction (10).

Oxidative stress (OS) is a prevalent pathological phenomenon in women afflicted with PCOS, as numerous studies demonstrated higher levels of ROS in women with PCOS compared with their healthy counterparts (14). This pathological condition can, in turn, increase the risk of OS-related diseases, such as cardiovascular disorders (15). The overexpression of ROS in OS conditions influences the quality of oocytes and ultimately lowers the rate of successful fertility (16). Decreased number of oocytes and poor oocyte quality are the main challenging factors in the fertility process, especially for the patients who need to undergo *in-vitro* fertilization (IVF) (17). Therefore, it seems necessary to use medications or therapeutic approaches to preserve oocytes in optimum status. As mentioned above, situin3, as an intracellular factor, can neutralize the deleterious effects of oxidative stress and regulate the development of early embryos by modulating the expression of crucial genes involved in this process. In the present study, we compared the effects of two commonly used ovulation-inducing drugs, metformin, and CC, on the expression of sirtuin3 in oocytes, collected from the mice, induced by PCOS.

## Experimental

*Ethical statement*

All procedures conducted in current study were in accordance with the National Institutes of Health Guidelines (NIH). The study was approved by ethical committee of Kharazmi University with code: 97/6505.

*PCOS induction and treatment protocols*

For the induction of PCOS in the mice, 24 female NMRI mice with the age of 6 weeks old were transferred to the laboratory and kept in standard conditions with free access to food (Behparvar, Iran) and water. Six healthy mice were also selected as the control group, and the rest received estradiol valerate (40 mg/kg; Aburaihan pharmaceutical Co., Iran) by the intraperitoneal injection to induce PCOS. Sixty days after the injection of estradiol valerate, the induction of PCOS was confirmed by the vaginal smear, and PCOS mice were divided into three experimental groups as follows: one group was kept untreated (sham), while two groups were treated with metformin and clomiphene. Metformin was administrated orally at a dose of 500 mg/kg for 20 days (18), and CC was administrated orally at a dose of 18 mg/kg for 10 days (19).

*Tissue processing and staining*

At the end of the treatment courses, the animals were sacrificed, and the left ovaries were fixed for pathological examinations. Briefly, ovaries were fixed in Bouin’s solution for 24 h and then dehydrated using the ascending concentrations of ethanol (Merck, Germany). In the next step, the tissues were cleared using toluene ((Merck, Germany) and then paraffin-embedded. The ovarian tissues were sectioned at a thickness of 5µm using a rotary microtome and then mounted on slides. For the staining process, tissue sections were deparaffinized by toluene and then rehydrated using the descending concentrations of ethanol. Finally, hematoxylin-eosin staining was carried out, and the sections were imaged under a light microscope to detect the presence of the ovarian cysts.

*Serum collection and hormonal analyzing*

For the collection of the serum samples, the whole blood was drawn from the heart of the animals and then left at room temperature for 30 min to clot. Subsequently, the samples were centrifuged at 1500 rpm for 10 min to separate the serum. The collected sera were kept at-80 °C until the hormonal analysis. The serum concentrations of FSH and LH were measured and compared among the four groups of the study. The levels of hormones were determined by commercial ELISA kits (Padtangostar Co.; Iran) according to the manufacturer’s recommendations. 

*Oocyte collection*

For the collection of oocytes, the ovaries were digested in a petri dish containing the Human Tubal Fluid (HTF) medium (Sigma Aldrich, UK) and then treated with the hyaluronidase (Sigma Aldrich, UK) enzyme for 10 sec to separate granulosa cells. Next, by means of a light microscope, the oocytes were gathered for RNA extraction. 

*RNA extraction and quantitative real-time PCR*

RNA extraction was performed in accordance with the RNA extraction kit (QIAGEN, Germany). Briefly, 300 µL of the Trizol (QIAGEN, Germany) reagent was added to microtube containing oocytes and kept at-80 °C for 24 h. Afterward, the pellets formed in the microtubes were crushed gently, and then 100 µL chloroform (Merck, Germany) was added. After one minute, the solution was centrifuged at 12,000 pm for 10 min, and the supernatants were collected. Then, cold isopropanol was added to microtubes to precipitate the RNA content of the cells. After washing the samples with ethanol, RNA was dried and recovered by DEPC (Sigma-Aldrich, UK) water. The extracted RNA was then converted into complementary DNA (cDNA) using the RevertAid cDNA Synthesize Kit (Fermentas, Canada).

The qRT-PCR reactions were performed in a final volume of 20 µL containing 10 µL SYBR Green master mix (Qiagen, Germany), 1 µL of each primer, 1 µL of cDNA, and 7 µL DEPC water. The thermal cycling was conducted based on the following program: initial denaturing step at 94 ˚C for 20 seconds, followed by 40 cycles at 60 ˚C for 30 sec and 72 ˚C for 30 sec. The GAPDH gene was used as the internal control, and the relative gene expression was determined using the 2^(-(∆∆CT)) ^method. The sequences of the primers (Kiagene, Iran) used in this study are listed in (Table 1).

*Statistical analysis*

All experiments were repeated at least three times, and the sample size was considered to be six animals per group. The obtained values were analyzed by Two-way analysis of variance (ANOVA), and the *P*-value of less than 0.05 was statistically significant. The data were represented as mean ± standard deviation. The SPSS.22 software was used to statistical analysis.

## Results

*Pathological examination of ovarian tissues*

**Figure 1:**
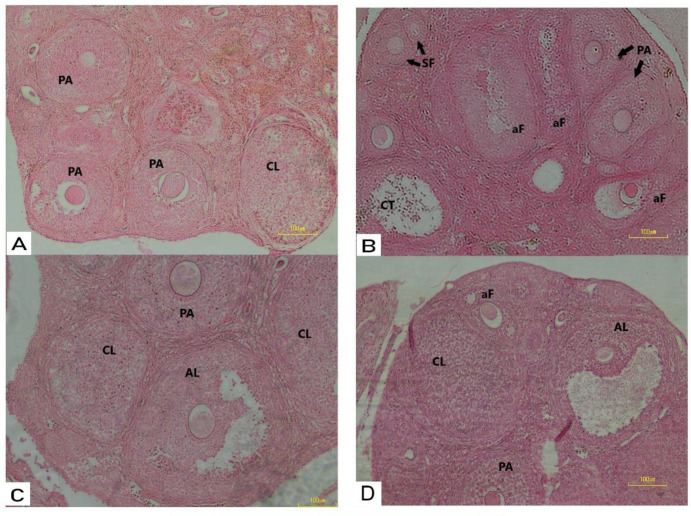
(A) Histological analysis of mice ovaries Control mice ovary showing normal follicles and corpus luteum; (B) PCOS mice ovary treated with estradiol valerate showing degeneration in follicles at development stages (atresia); (C) Photomicrograph of mice ovary treated with E.V plus CC showing more active ovary than PCOS with follicles as well as corpora lutei, preantral follicle and antral follicle; (D) Mice ovary treated with E.V plus metformin showing follicles in different developmental stages, as well as corpus luteum. SF: secondary follicle; PA: pre-antral follicle; AL: antral follicles; aF: atratic follicle: CL: corpus luteum. (H&E x20)

Tissue sections obtained from all experimental groups were examined to analyze the histological changes to detect the presence of ovarian cysts. The histopathological observations revealed that the injection of estradiol valerate successfully induced PCOS in mice and caused syndrome-related changes, such as the formation of hydatid cysts, a reduction in the numbers of primary, secondary, and antral follicles, as well as the absence of the corpus luteum. The results also demonstrated that the administration of metformin or clomiphene significantly diminished the deleterious effects of PCOS induction treated animals, and the ovulation process was mostly restored, as shown by the presence of the corpus luteum and pre-antral follicles in tissue sections (Figure 1). 

**Figure 2:**
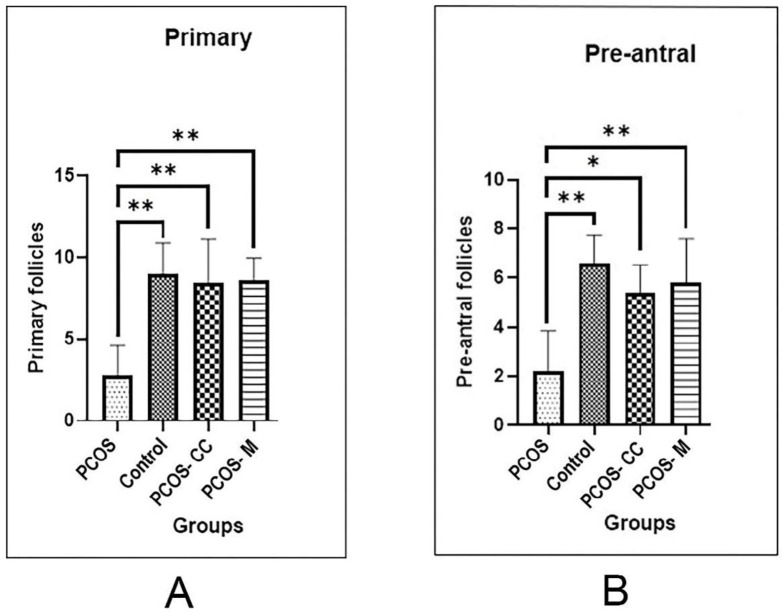
Comparison the mean of follicle count between control, PCOS, and treatment groups (A) Primary follicle, (B) Pre-antral follicles. PCOS: polycystic ovary syndrome, control: healthy control, PCOS-CC: polycystic ovary syndrome treated with CC, PCOS-M: polycystic ovary syndrome treated with metformin. (Mean ± SD; ns: P > 0.05, *: P ≤ 0.05, **: P ≤ 0.01, ***: P ≤ 0.001

A significant increase was found in the number of pre-antral follicles in metformin (mean number = 5.8 ± 1.7; *P* < 0.05) and CC-treated (mean number = 5.4± 1.1; *P* < 0.05) groups when compared with the sham group (mean number = 2.2 ± 1.6; *P* < 0.05) (the mice induced by PCOS and left untreated) (*P* < 0.05). The results indicated that the frequency of primary follicles was higher in all experimental groups than the PCOS group. A significant increase was detected in the number of primary follicles in PCO + metformin (mean number = 8.6 ± 1.3; *P* < 0.05), PCOS + CC (mean number = 8.4 ± 2.7; *P* <0.05) groups when compared with the PCOS group (mean number = 2.8 ± 1.7; *P* < 0.05). No significant difference was observed in the number of primary and pre-antral follicles between the experimental groups and the control group (*P* ˃ 0.05) (Figure 2).

*Serum levels of FSH and LH*

**Figure 3:**
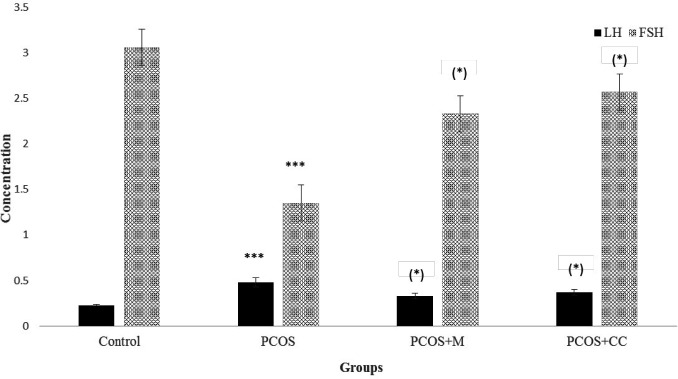
Serum LH (ng/mL) and FSH (mIU/mL) levels in different groups. Control: healthy control, PCOS: polycystic ovary syndrome, PCOS+M: polycystic ovary syndrome treated with metformin, PCOS + CC: polycystic ovary syndrome treated with CC. Star in bracelet: in compare to PCOS group, stars without bracelet: in compare to control. (Mean ± SD, ***: P < 0.001; *: P < 0.05).

The results indicated that the FSH level (mIU/mL) was significantly lower in the sham group (1.4 ± 0.4) than the control group (3.06 ± 0.4), whereas the level of LH (ng/mL) was considerably higher in sham (0.5 ± 0.1) compared to the control (0.24 ± 0.02). All differences were significant at *P* < 0.001. Also, a marked increase was shown in the ratio of LH to FSH in the sham group (0.357) compared with the control group (0.075). The administration of metformin and clomiphene returned the changed sex hormonal levels of the mice induced by PCOS to the baseline values; so, there was not any significant difference in the level of FSH and LH between control and medication treated animals. The level of FSH in metformin treated animals was 2.35 ± 0.5 and in CC treated animals was 2.6 ± 0.5. Serum LH in metformin and CC treated animals was 0.35 ± 0.05 and 0.4 ± 0.05, respectively. The ratio of LH to FSH was reduced significantly in metformin (0.14) and CC (.0143) treated animals compared to PCOS animals (*P* < 0.05) (Figure 3).

*Expression of Sirt3 gene*

**Figure 4:**
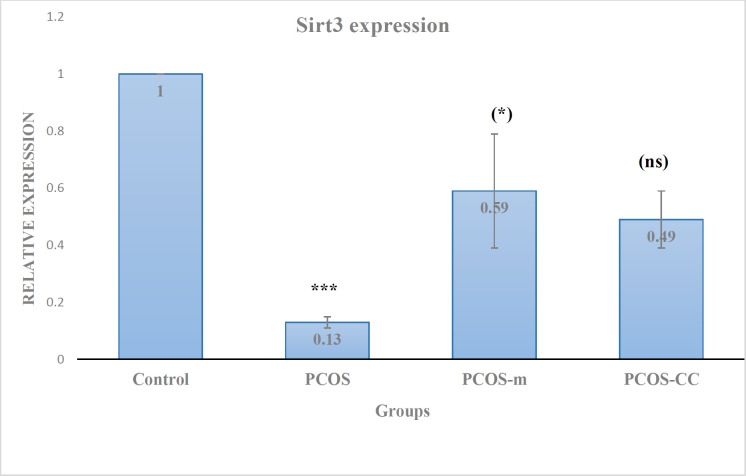
Relative expression of Sirt3 gene. Control: healthy control; PCOS: polycystic ovary syndromes; PCOS-m: polycystic ovary syndrome treated with metformin; PCOS-cc: polycystic ovary syndrome treated with CC. Star in bracelet: in compare to PCOS group; stars without bracelet: in compare to control; (ns): non-significant in compare to PCOS group. (Mean ± SD; *: P < 0.05, ***: P < 0.001)

The results of the gene expression analysis showed that Sirt3 expression was significantly reduced by 10 folds in the sham group in comparison with the control group, so the relation expression of Sirt3 gene in PCOS group was 0.13 compared to the control (*P* < 0.001). The administration of metformin significantly increased the expression of Sirt3 in oocytes of the metformin-treated mice in comparison with oocytes obtained from the sham group; the relation expression of Sirt3 gene in PCOS + metformin group was 0.59 which is not significantly different with control but at *P* < 0.05, it was different with PCOS group. The administration of clomiphene reached the expression of Sirt3 gene to 0.49 in relation to the control, indeed CC did not significantly alter the expression of Sirt3 in CC-treated PCOS mice compared to the PCOS animals and *P*-value was > 0.05. No significant difference was found in the expression of Sirt3 between CC-and metformin-treated mice that may be due to a slight increase in the expression of Sirt3 in the mice receiving clomiphene (Figure 4).

## Discussion

Oocytes are specialized cells that are enriched by mitochondria. A high rate of ROS production in these organelles makes them susceptible to damages, which, in addition to the risk of mutation in mitochondrial DNA, also severely affects the fertility potential of the oocytes (20). Proper mitochondrial activity is critical for implantation and early stages of embryo development (21). Although physiological levels of ROS (60 ng/oocyte) would be a requisite during oocyte maturation, increased ROS generation in oocytes was associated with impaired fertilization and early embryogenesis in a mouse model of obesity (16, 22).

In the present study, the impact of metformin and clomiphene citrate on the expression of the Sirt3 gene was investigated. The results showed that metformin prevented the reduction of Sirt3 expression in oocytes derived from the mice induced by PCOS. In other words, metformin decreased the serum level of LH and made a balance in the ratio of LH to FSH. It also stimulated the ovulation process in the ovary of the mice with PCOS and regulated the decreased expression of Sirt3 in oocytes. This mechanism of action was identified for the first time in our study, and it appears to result from the ability of metformin to suppress oxidative stress-related factors in the cells. Chakraborty and colleagues (2011) investigated 208 patients with type 2 diabetes and showed that the administration of metformin could reduce oxygen free radicals and increase the power of the antioxidant system in these patients (23). Kocer *et al*. (2014) demonstrated that in women with PCOS, the serum level of malondialdehyde (MDA) is significantly decreased when treated with metformin (24). MDA is a toxic product of aldehydes, and it is a remarkable biomarker of oxidative stress (25). 

The role of metformin in preventing the reduction of Sirt3 expression can contribute to the enhancement of the oocyte quality for the fertilization process. Zhao *et al*. delineated the role of Sirt3 in oocyte aging in 324 infertile women. They compared the expression levels of Sirt3 in oocytes in *in-vitro* maturation (IVM) and *in-vivo* maturation conditions. They showed that the expression rate of Sirt3 is lower in an IVM condition, implying a higher rate of ROS production and subsequently oocyte aging and impaired development potential (26). Their result was in line with the previous findings of Kawamura, *et al*., who indicated that knockdown of Sirt3 expression by siRNA in oocytes could lead to reduced development efficiency (27).

Although CC is one of the first-line therapeutic options in inducing the ovulation process for patients with PCOS, it has considerable limitations for those with the body mass index (BMI) > 30. In agreement with this statement, a study conducted by Legro, *et al*. found a significant difference in pregnancy rates between patients with BMI > 30 and those with BMI < 30 (28). It has been shown that obesity can disrupt the function of mitochondria and is a major cause of Sirt3 reduction in oocytes of obese mice (7). According to our finding, the clomiphene inability in obese women to induce qualified oocytes for fertilization, may be explained with clomiphene inability to prevent the decrease in expression of Sirt3 in oocytes.

For elucidating the mechanisms of action of Sirt3 in preventing oxidative stress, one study performed on diabetic mice showed that Sirt3 leads to the removal of oxygen free radicals by acetylation of the superoxide dismutase enzyme (29). Besides, gain-and-loss studies conducted on human oocytes revealed that impaired expression of Sirt3 disrupts the biogenesis of mitochondrial DNA, resulting in the disruption of oocyte development (26). 

**Table 1:** Primers used for real-time PCR analyses

**Primer name**	**Sequence**	**Product Length**
**Sirt3**	Forward: CGGCTTTGGAGGTGGAGGAAG Reverse: GAAAAAGGGCTTGGGGTTGTGA	247 bp
**GAPDH**	Forward: ATGACATCAAGAAGGTGGTGAAG Reverse: GAAGGTGGAAGAGTGGGAGTTG	119 bp

## Conclusion

The present study showed that the induction of PCOS reduced the expression of Sirt3 in oocytes, showing one of the mechanistic pathological features of PCOS in the development of infertility. A comparison of the effects of metformin and clomiphene on Sirt3 expression in oocytes that were obtained from the mice, induced by PCOS, showed that metformin is capable of preventing the downregulation of Sirt3, while clomiphene did not have a role in this regard. This finding signifies the impact of ovulation-inducing drugs on the quality of oocytes in the fertility process and can be taken into account to understand the mechanism of action of these drugs in future studies.
